# Assessment of dissociation among combat-exposed soldiers with and without posttraumatic stress disorder

**DOI:** 10.3402/ejpt.v6.26657

**Published:** 2015-04-28

**Authors:** Barbaros Özdemir, Cemil Celik, Taner Oznur

**Affiliations:** Department of Psychiatry, Gülhane Military Medical Faculty, Ankara, Turkey

**Keywords:** Combat exposure, posttraumatic stress disorder, dissociation, dissociative symptoms, physical injury

## Abstract

**Background:**

Dissociation is a disruption of and/or discontinuity in the normal, subjective integration of one or more aspects of psychological functioning, including memory, identity, consciousness, perception, and motor control. A limited number of studies investigated combat-related dissociation.

**Objective:**

The primary aim of this study was to evaluate the relationship between dissociative symptoms and combat-related trauma.

**Method:**

This study included 184 individuals, including 84 patients who were exposed to combat and diagnosed with posttraumatic stress disorder (PTSD) (Group I), 50 subjects who were exposed to combat but were not diagnosed with PTSD (Group II), and 50 healthy subjects without combat exposure (Group III). The participants were evaluated using the Dissociative Experiences Scale (DES) to determine their total and sub-factor (i.e., amnesia, depersonalization/derealization, and absorption) dissociative symptom levels. In addition, Group I and Group II were compared with respect to the relationship between physical injury and DES scores.

**Results:**

The mean DES scores (i.e., total and sub-factors) of Group I were higher than those of Group II (*p*<0.001), and Group II's mean DES scores (i.e., total and sub-factors) were higher than those of Group III (*p*<0.001). Similarly, the number of subjects with high total DES scores (i.e.,>30) was highest in Group I, followed by Group II and Group III. When we compared combat-exposed subjects with high total DES scores, Group I had higher scores than Group II. In contrast, no relationship between the presence of bodily injury and total DES scores could be demonstrated. In addition, our results demonstrated that high depersonalization/derealization factor scores were correlated with bodily injury in PTSD patients. A similar relationship was found between high absorption factor scores and bodily injury for Group II.

**Conclusions:**

Our results demonstrated that the level of dissociation was significantly higher in subjects with combat-related PTSD than in subjects without combat-related PTSD. In addition, combat-exposed subjects without PTSD also had higher dissociation levels than healthy subjects without combat experience.

Dissociation is the process through which individuals experience a disruption or discontinuity in one or several aspects of their psychological functioning. The functions that are affected by dissociation typically include memory, perception, consciousness, identity, and motor control (Candel & Merckelbach, 2004). Dissociation symptoms are believed to play a significant role in the pathogenesis and continuation of posttraumatic stress disorder (PTSD) and similar conditions (Wolf et al., 2012). The relationship between PTSD and dissociation is a clinically important subject; however, there is currently no consensus regarding the nature of this relationship.

Researchers have proposed different explanations concerning the relationship between PTSD and dissociation. Waelde, Silvern, and Fairbank (2005) performed a taxometric analysis of the Dissociative Experiences Scale (DES) scores of Vietnam War veterans who had been exposed to trauma and determined that 32% of the veterans with PTSD also had discrete dissociation. Putnam, Carlson, Ross, and Anderson (1996) indicated that the mean DES scores of PTSD patients were a function of a subset of participants with very high dissociation levels rather than a representation of a continuous dispersion of similar symptoms within the group. Similarly, based on an evaluation of existing neurobiological and psychometric studies, Lanius et al. (2010) identified evidence for a dissociative subtype of PTSD. The alternative to the dissociative subtype hypothesis is that dissociation represents a primary facet of PTSD that has an equal distribution among patients with PTSD and that dissociation is a general marker for the severity of the condition (Van der Kolk, Pelcovitz, Roth, & Mandel, 1996). This distinction has various implications for the revision of the Diagnostic and Statistical Manual of Mental Disorders, Fifth Edition (DSM-5) criteria for PTSD. The DSM-5 includes a dissociative subtype for PTSD, which is described as being characterized by prominent depersonalization (i.e., detachment or feelings of unreality regarding one's self) and derealization (i.e., detachment or feelings of unreality regarding one's environment) (DSM-5; American Psychiatric Association, 2013; Blevins, Weathers, & Witte, 2014).

This revision reflects a long history of clinical observations and studies that identified a relationship between trauma and dissociation. This revision also takes into account neurobiological studies that demonstrated that higher levels of differential response patterns occur among PTSD patients who have predominantly re-experiences in comparison to patients who have a dissociative response to trauma (Carlson, Dalenberg, & McDade-Montez, 2010; Lanius, Brand, Vermetten, Frewen, & Spiegel, 2012).

The main goal of the current study was to evaluate the existing data and information regarding dissociative symptoms among patients with combat-related PTSD. Some previous studies demonstrated that high levels of dissociative symptoms occur among veterans with combat-related PTSD (Elhai, Frueh, Davis, Jacobs, & Hamner, 2003; Hyer, Albrecht, Boudewyns, Woods, & Brandsma, 1993; Loewenstein & Putnam, 1988; Marmar et al., 1994; Stutman & Bliss, 1985; Spiegel, Hunt, & Dondershine, 1988). Many of these studies focused on determining why some military veterans who are exposed to combat-related trauma develop dissociative symptoms while others who experience similar trauma do not exhibit such symptoms. In addition, widespread evidence indicates that PTSD patients experience considerably higher levels of dissociative experiences than individuals without PTSD (Bremner & Brett, 1997; Bremner, Steinberg, Southwick, Johnson, & Charney, 1993; Bremner et al., 1992; Keane, Kaufman, & Kimble, 2001). Thus, these studies indicate that dissociation may be a significant factor that influences the relationship between PTSD development and PTSD symptoms (Suliman, Troeman, Stein, & Seedat, 2013). The importance of the relationship between trauma and dissociation was highlighted again by the identification of a dissociative type of PTSD in the DSM-5.

Our study is important because it includes healthy individuals with no history of combat-related trauma as a comparison group when assessing the level of dissociation among soldiers with and without PTSD who were exposed to war-related trauma. Furthermore, this study aimed to demonstrate that high dissociation levels are associated with an increased risk of developing combat-related PTSD. Previous studies that investigated the relationship between PTSD symptoms and physical injuries found that high-level dissociation is positively correlated with physical injury (Koren, Norman, Cohen, Berman, & Klein, 2005; Kulka et al., 1990; Pitman, Altman, & Macklin, 1989). The current study also evaluated the relationship between physical injury and the level of dissociation. However, our findings showed that the increase in some dissociative symptoms (i.e., depersonalization–derealization) in patients with physical injury was specific to combat-related PTSD patients; this finding is significant and distinguishes our study from previous studies.

## Method

### Participants

Male subjects aged between 20 and 40 who were currently serving in the Turkish Armed Forces as professional soldiers (i.e., officers, sergeants and other mercenaries) and had a long history of compulsory service (i.e., between 10 and 15 years) were included in the study. Group I consisted of 84 subjects who had experienced combat and were diagnosed with PTSD, while Group II consisted of 50 subjects who had experienced combat but were not diagnosed with PTSD. The combat experiences of the participants resulted in traumatic events during battle with terrorist organizations. Group III included 50 healthy subjects with no combat experience. The subjects were evaluated by two psychiatrists, who diagnosed the subjects in Group I with PTSD according to the DSM-IV. In addition, the subjects in this group were required to have had no Axis I disorder diagnosed during the 6 months preceding the study. Sixteen participants who had a comorbid Axis I disorder in the past 6 months were excluded from study. A single psychiatrist performed the evaluations for the subjects in Group II and Group III. Based on this evaluation, subjects with a DSM-IV Axis I disorder, a central nervous system disease, a metabolic disorder, mental retardation, or substance dependence were not included in the study. None of the subjects had used alcohol, any illicit substance, or medication during the 4 weeks preceding the study. All subjects were thoroughly informed about the study procedures. Approval for the study protocol was obtained from the Ethics Committee of the Gülhane Military Medical Faculty.

### Assessments

Demographic information (e.g., age, education level, and marital status) and combat-experience-related characteristics (e.g., type and duration of combat and physical injuries) were recorded during the psychiatric interview. These data were verified by consulting the official documents and medical records of the Turkish Army. PTSD was diagnosed using the Turkish version of the Structured Clinical Interview for the DSM-IV Axis I Disorders (SCID-I) Form (First, Spitzer, Gibbon, & Williams, 1997; Özkürkçügil, Aydemir, Yıldız, Danacı, & Köroğlu, 1999). Dissociation was evaluated using the Turkish version of the DES, which is a 28-item self-report measure that assesses the frequency of dissociative experiences, such as amnesia, gaps in awareness, depersonalization, derealization, absorption, and imaginative involvement (Bernstein & Putnam, 1986; Yargıç, Tutkun, & Sar, 1995). The total DES scores and the three main factor scores (i.e., amnesia, depersonalization and derealization, and absorption) were calculated by averaging the scores for all or related items. A DES score greater than 30 is considered to be the threshold point for dissociative disorders in the Turkish population (Yargıç et al., 1995).

### Statistical analysis

Statistical analyses were performed using SPSS software, version 16. The variables were investigated using visual (i.e., histograms and probability plots) and analytical methods (i.e., Kolmogorov–Smirnov/Shapiro–Wilk tests) to determine whether or not the variables were normally distributed. Descriptive statistics were expressed as the mean±standard deviation and the percentage. Group comparisons were performed using the Chi-square test and ANOVA, where applicable. The Tukey test was used to perform *post-hoc* evaluations. As parametric conditions were not normally distributed, the Kruskall–Wallis test was performed to compare the DES scores of the three groups. The Mann–Whitney *U* test was performed to test the significance of pairwise differences, using the Bonferroni correction to adjust for multiple comparisons. The Friedmann test was used to determine the effect of the DES sub-scores on the total DES scores. The Wilcoxon test was performed to test the significance of pairwise differences, using the Bonferroni correction to adjust for multiple comparisons. Logistic regression analysis was employed to elucidate the determinant factors among combat-exposed subjects with DES scores >30. *p*-Values >0.05 were considered statistically significant.

## Results

This study included 184 participating subjects (Group I: 84, Group II: 50, and Group III: 50). The three groups were similar with respect to sociodemographic characteristics (Table 1). The DES scores of the participants are provided in Tables 2 and 3. The mean overall DES scores (i.e., total and sub-factors) of Group I were comparatively higher than those of Group II (*p*<0.001), while Group II's mean overall DES scores were higher than those of Group III (*p*<0.001). Similarly, the number of subjects with a total DES score >30 differed significantly among the groups, as follows: Group I>Group II>Group III (*p*<0.001). In addition, subjects who had total DES scores>30 were compared with respect to overall DES scores (Table 2). In Group I, the depersonalization/derealization sub-factor scores were higher than the other two sub-factor scores (i.e., amnesia and absorption) (*p*<0.001). A significant difference between the absorption sub-factor scores and the other sub-factors scores was observed in Group II (*p*<0.001) (Table 3). Groups I and II were similar with respect to bodily injury and other combat-related characteristics (i.e., armed conflict, mine, or bomb effects) (Table 4). The factors that affected the DES scores are listed in Table 5. The presence of PTSD was identified as the only factor that was significantly related to the DES scores of the subjects. This factor was also associated with the number of subjects with a DES score>30. In contrast, no relationship between the presence of bodily injury and the total DES scores could be demonstrated. Furthermore, our results demonstrated that high depersonalization/derealization factor scores were correlated with bodily injury among PTSD patients. A similar relationship was found between high absorption factor scores and bodily injury for Group II (Table 6).

**Table 1:** Sociodemographic features of the subjects

Features	Group I (*n*=84)	Group II (*n*=50)	Group III (*n*=50)	Test statistic	*p*
Age (years)^a^	30.3±5.6	29.0±5.0	28.8±4.0	1.8	0.17
Education (years)^a^	9.5±2.0	8.5±3.3	8.7±3.4	2.4	0.09
Marital status, *n* (%)^b^					
Married	46 (54.8)	22 (44)	15 (30)	8.2	0.08
Single	28 (33.3)	22 (44)	28 (56)		
Divorced	10 (11.9)	6 (12)	7 (14)		

Group I: Combat-exposed subjects with PTSD; Group II: Combat-exposed healthy subjects; Group III: Healthy subjects.

a ANOVA

b Chi-Square test

**Table 2:** Comparison of Dissociative Experiences Scale (DES) scores among groups

Features	Group I	Group II	Group III	Test statistic	Comparisons	*p*
DES scores [*n* (%)]	84 (45.6)	50 (27.2)	50 (27.2)			
Total score [mean (SD)]^a^	44.4 (19.9)	26.9 (12.4)	13.3 (13.3)	81.8	I>II>III	<0.001*
DES Amn score [mean (SD)]^a^	13.7 (6.6)	9.3 (4.4)	4.2 (3.8)	75.5	I>II>III	<0.001*
DES Dep/Der score [mean (SD)]^a^	17.1 (7.8)	7.9 (3.4)	4.2 (4.5)	94.9	I>II>III	<0.001*
DES Abs score [mean (SD)]^a^	13.4 (6.5)	9.4 (4.8)	4.9 (5.2)	62.0	I>II>III	<0.001*
DES score>30 [*n* (%)]^b^	59 (71.9)	19 (23.2)	4 (4.9)	50.3	I>II>III	<0.001*
Total score [mean (SD)]^a^	53.8 (15.3)	39.6 (7.7)	50.0 (14.7)	12.0	I=III>II	0.002*
DES Amn score [mean (SD)]^a^	16.6 (5.6)	13.5 (3.5)	13.7 (4.7)	6.0	I>II=III	0.049*
DES Dep/Der score [mean (SD)]^a^	20.8 (5.9)	11.3 (2.1)	17.0 (3.8)	35.9	I=III>II	<0.001*
DES Abs score [mean (SD)]^a^	16.2 (5.5)	14.2 (3.6)	19.2 (6.6)	3.0	–	0.21

Group I: Combat-exposed subjects with PTSD; Group II: Combat-exposed healthy subjects; Group III: Healthy subjects.

DES Amn: DES amnesia factor; DES Dep/Der: DES depersonalization/derealization factor; DES Abs: DES absorption factor.

a Kruskal–Wallis test.

b Chi-square test value.

* Statistically significant.

**Table 3:** Effect of Dissociative Experiences Scale (DES) sub-factor scores on total DES scores

	DES Dep/Der score [mean (SD)]	DES Amn score [mean (SD)]	DES Abs score [mean (SD)]	Test statistic^a^	*p*
Group I (*n*=84)	17.1 (7.8)^b^	13.7 (6.6)	13.4 (6.5)	48.3	<0.001*
Group II (*n*=50)	7.9 (3.4)^b^	9.3 (4.4)	9.4 (4.8)	22.2	<0.001*
Group III (*n*=50)	4.2 (4.5)	4.2 (3.8)	4.9 (5.2)	4.9	0.085

DES Amn: DES amnesia factor; DES Dep/Der: DES depersonalization/derealization factor; DES Abs: DES absorption factor.

* Statistically significant.

a Friedman test value.

b Factors that affect the total DES score.

**Table 4:** Comparison of Groups I and II with respect to combat experiences (i.e., bodily injury, traumatic events, and posttraumatic duration since trauma)

	Group I (*n*=84)	Group II (*n*=50)	Test statistic	*p*
Posttraumatic duration (months), mean (SD)	47.2 (28.3)	53.7 (23.8)	−1.2^a^	0.23
Traumatic event, *n* (%)				
Mine explosion	8 (9.5)	3 (6)	3.1^b^	0.21
Grenade explosion	4 (4.8)	0 (0)		
Shootout	72 (85.7)	47 (94)		
Bodily injury, *n* (%)				
Yes	20 (23.8)	8 (16)	1.2^b^	0.28
None	64 (76.2)	42 (84)		

Group I: Combat-exposed subjects with PTSD; Group II: Combat-exposed healthy subjects;

a Mann–Whitney *U* test, Z value.

b Chi-square test value.

**Table 5:** The relationship between DES scores and certain factors among subjects in Groups I and II with a total DES score>30

	Beta	Relative risk	95% CI	*p*
Age (years)	0.04	1.04	0.97–1.13	0.28
Education (years)	0.02	1.02	0.88–1.19	0.78
Marital status	0.05	1.05	0.56–1.95	0.89
Bodily injury type	0.66	0.52	0.22–1.20	0.12
Presence of injury	−0.88	0.42	0.15–1.15	0.09
Posttraumatic duration	0.37	1.45	0.85–2.48	0.17
Presence of PTSD	−1.25	0.29	0.12–0.66	0.003*

Group I: Combat-exposed subjects with PTSD; Group II: Combat-exposed healthy subjects.

* Statistically significant.

**Table 6:** The relationship between DES scores and bodily injury

	Group I (*n*=84)				Group II (*n*=50)			
				
Features	BI (*n*=20)	nBI (*n*=64)	*Z*	*p*	BI (*n*=8)	nBI (*n*=42)	*Z*	*p*
Total DES scores	44.9±17.2	44.2±20.8	−0.21	0.833	36.1±17.7	25.1±10.4	−1.92	0.055
DES Amn	11.2±5.1	14.5±6.8	−1.90	0.058	11.5±6.7	8.8±3.7	−1.13	0.258
DES Dep/Der	22.3±7.4	15.5±7.3	−3.05	0.002*	10.0±4.6	7.5±3.0	−1.56	0.119
DES Abs	11.3±5.4	14.0±6.7	−1.54	0.123	13.3±6.9	8.7±4.0	−2.06	0.040*

Group I: Combat-exposed subjects with PTSD; Group II: Combat-exposed healthy subjects;

BI: Bodily injury-positive; nBI: Bodily injury-negative; DES Amn: DES amnesia factor.

DES Dep/Der: DES depersonalization/derealization factor; DES Abs: DES absorption factor.

Z: Mann–Whitney *U* test value.

* Statistically significant.

## Discussion

In this study, the dissociative symptoms of both healthy subjects and combat-exposed soldiers with and without PTSD were evaluated. The study results revealed higher levels of dissociation among combat-exposed subjects with PTSD than among combat-exposed subjects without PTSD. Combat-exposed subjects without PTSD also had higher dissociation levels than healthy subjects without combat experience.

The relationship between dissociation and traumatic experiences has been investigated primarily among World War II and Vietnam veterans (Banscomb, 1991; Bremner et al., 1992; Elhai et al., 2003; Hyer et al., 1993). Bremner et al. (1992) demonstrated previously that for combat-exposed Vietnam War veterans, the levels of dissociation are higher among those with PTSD than among those without PTSD. Those authors also observed that higher dissociation levels were related to the development of PTSD. The results of our study were similar; however, our study also included a control group and demonstrated that combat-exposed soldiers without PTSD had higher levels of dissociation than healthy subjects without combat experience. Thus, combat exposure/experience alone appears to lead to an increase in the level of dissociation. Bremner et al. (1992) reported a mean DES score of 13.7 among soldiers without PTSD or any other psychiatric disorder. The scores reported by Bremner et al. (1992) were lower than the scores observed in our study. This difference may be due to the samples, as our study groups consisted of soldiers who had a long history of compulsory service. Thus, these soldiers may have experienced more traumatic events during their long compulsory service period. Furthermore, in the studies described above, the DES was applied long after the soldiers had left the traumatic environment (e.g., more than 30 years after the Second World War or the Vietnam War). Therefore, distance and separation in time might have affected the DES scores. Another explanation involves differences between the countries with respect to beliefs and sociocultural features (Tutkun et al., 1998). Yargıç et al. (1995) reported that dissociative findings were common among the Turkish population.

Briere et al. demonstrated that persistent dissociation, peritraumatic dissociation, peritraumatic distress, and generalized dissociative symptoms are all associated with PTSD. Similar to our study, the level of generalized dissociative symptoms was measured using the DES in the study performed by Briere et al. (Briere, Scott, & Weathers, 2005; Briere, Weathers, & Runtz, 2005). Van der Kolk et al. (1996) previously determined that patients who met the criteria for lifetime PTSD (but not current PTSD) had dissociation levels that were significantly lower than those of patients with current PTSD but significantly higher than those of patients without PTSD. The results of our study were similar to those of Bremner and Wolf, as they also revealed that higher generalized dissociation levels are associated with PTSD. The main feature that distinguishes our study from the study of Bremner and Wolf is that we also included healthy soldiers who had experienced no combat-related trauma. Many psychiatric patients and normal subjects may report dissociative experiences. In general, the mean DES scores of subjects with traumatic lifestyles are higher than those of the general population. Ross, Joshi, and Currie (1988) previously reported a mean DES score of 10.8 for the general population. In Turkey, this score has been reported to be 7.67 (Akyuz, Dogan, Sar, Yargic, & Tutkun, 1999). Soldiers have higher levels of dissociation than many other occupational groups, primarily because soldiers are exposed to much higher levels of stress and experience traumatic events more frequently. Even soldiers who did not experience traumatic events report higher DES scores than normal subjects (Gulsum, Özdemir, Celik, Uzun, & Ozsahin, 2009). In this study, we determined that DES scores (13.3) were higher among healthy soldiers than among the normal population. This observation is consistent with the findings of previous studies (Ross et al., 1988). Our study results also demonstrated that the levels of dissociation of soldiers with PTSD who experienced traumatic events were higher than those of soldiers without PTSD who experienced similar traumatic events. Thus, the dissociation levels of PTSD patients were determined to be higher than those of individuals who did not develop PTSD, despite exposure to combat-related stressors. Our study results also demonstrated that the levels of dissociation remained high among these soldiers. Based on these observations, it is possible to conclude that while low dissociation levels occur as part of a defense mechanism against stressors, high dissociation levels in response to stress play an important role in the development of PTSD (Bowins, 2004; Briere, Scott, et al., 2005).

In the literature, the number of studies that evaluated the relationship between dissociation and physical injury during deployment is somewhat limited. Traditional views, particularly psychoanalytic views, tended to regard bodily injury as a protective factor against the development of PTSD (Ulman & Brothers, 1987). At the core of this assumption is the theory that physical injury absorbs much of one's “free-floating psychic energy,” thus reducing the chance of developing anxious or conflicting feelings about the traumatic event. In addition, unlike psychological wounds, bodily injuries typically engender more sympathy from the environment. However, over the past two decades, numerous studies of injured trauma survivors challenged this traditional understanding of the relationship between PTSD and injury (Kulka et al., 1990; Pitman et al., 1989). While dissociation is commonly viewed as a factor that increases vulnerability because it impairs the integration and reprocessing of the trauma, an interesting alternative possibility is that the relationship between bodily injury and dissociation might be reversed. Specifically, people who tend to dissociate under stress might be at higher risk of getting injured (Koren et al., 2005).

Various studies reported that the occurrence of physical injury during deployment is associated with an increased likelihood of developing PTSD (Hoge et al., 2008; Kennedy et al., 2007). Hoge et al. (2008) conducted a study of soldiers returning from deployment and demonstrated that while 9% of soldiers who did not sustain any injuries subsequently developed PTSD, this ratio increased nearly two-fold to 16% for those who were injured during deployment. A similar ratio was observed in a study that was conducted by Koren et al. (2005) and evaluated the increase in PTSD incidence that occurs due to combat-related injuries. That study included 60 injured and 40 uninjured soldiers and reported PTSD frequency values of 16.7 and 2.5%, respectively, for these two groups of soldiers. In addition, Koren et al. also reported higher levels of depression, anxiety, and dissociation among injured soldiers. Thus, physical injuries can play a significant role in the development of PTSD and other psychological disorders. Another post-deployment study identified a relationship between the number of injuries sustained by soldiers and the incidence of PTSD. Further, a previous study reported that the occurrence of one, two, and three or more instances that resulted in injury to soldiers were associated with PTSD incidence rates of 14, 29, and 51%, respectively (Schneiderman, Braver, & Kang, 2008). These results are consistent with the PTSD incidence rates observed among civilians who experienced major orthopedic trauma (McCarthy et al., 2003; Starr et al., 2004). Our study results indicated that there was no relationship between physical injury and total DES scores in PTSD and non-PTSD patients. In addition, our study results revealed a relationship between physical injury and depersonalization/derealization factor scores in Group I. A similar relationship was found between high absorption factor scores and bodily injury for Group II. These findings indicate that physical injury (rather than the total DES score) is associated with an increase in depersonalization/derealization levels in PTSD patients. In addition, these findings indicate the existence of a specific relationship between dissociation and physical injury in the context of PTSD. Furthermore, these findings may suggest an association between physical injury and the dissociative type of PTSD that was described in the DSM-5. The presence of a nearly significant difference between the total DES scores of patients with and without injury in Group II may be due to the effects of the absorption factor in this group. However, because we did not assess the severity of the subjects’ physical injuries before obtaining these findings and thus did not determine whether differences exist between the groups in terms of physical injury severity, the results could have been affected; this possibility is a limitation of the study design.

Our study has several other limitations. First, the size of our study population was relatively small. Second, the study subjects were not evaluated with respect to traumatic brain injury (TBI). Although it was once assumed that the loss of consciousness and the memory deficits that are associated with TBI render the development of PTSD unlikely, recent studies demonstrated that PTSD could develop even among patients who have no conscious memory of the traumatic event (Gil, Caspi, Ben-Ari, Koren, & Klein, 2005; Harvey, Brewin, Jones, & Kopelman, 2003; Klein, Caspi, & Gil, 2003; Warden et al., 1997). Studies among military personnel suggested that conditions that can potentially cause brain injury, such as TBI, are associated with a high PTSD incidence (Kennedy et al., 2007). A third limitation of our study was the lack of a group that included patients who had PTSD in association with traumatic events that were unrelated to combat. Some evidence suggests that veterans with PTSD caused by combat-related events experience more severe symptoms than veterans with PTSD caused by events unrelated to combat (Brinker, Westermeyer, Thuras, & Canive, 2007). A fourth limitation of our study was the fact that the subjects were not evaluated for possible childhood trauma exposure, adversities, the length of combat exposure, and peritraumatic dissociation. Previous studies have indicated that psychiatric disorders, such as depression, anxiety disorders, and dissociative, somatoform, and psychotic disorders increase the level of dissociation. In addition, some neurological disorders (e.g., primarily epilepsy, migraine headache, and spinal cord injury) and organic disorders (e.g., hypoglycemia, uremia, and malaria) increase the level of dissociation (Putnam, 1985; Putnam et al., 1996). Therefore, participants with comorbid disorders were excluded from the study to clarify the effect of combat trauma on the dissociation level. Thus, we aimed to evaluate changes in dissociation levels in the context of PTSD psychopathology. Simultaneously, we also aimed to evaluate the effect of physical injury on the dissociation level independently from other factors. However, by excluding participants with comorbid disorders, we might have studied with participants who have lower dissociation levels than typical PTSD patients; this possibility is the fifth and final limitation of the current study.

## Conclusions

In conclusion, our study results demonstrated that the level of dissociation is higher among Turkish subjects with combat-related PTSD than among subjects without combat-related PTSD. In addition, combat-exposed subjects without PTSD also had higher dissociation levels than healthy subjects without combat experience. Further, our results showed that high depersonalization/derealization factor scores were correlated with bodily injury among PTSD patients. Future studies should investigate the effectiveness of various approaches for the treatment of high dissociation levels in larger samples of PTSD patients, preferably without any other significant psychiatric disorders. In addition, it is important for future studies to examine specific factors of dissociation rather than treating dissociation as a single construct.
